# Struggling for a feasible tool – the process of implementing a clinical pathway in intensive care: a grounded theory study

**DOI:** 10.1186/s12913-018-3629-1

**Published:** 2018-11-06

**Authors:** Petronella Bjurling-Sjöberg, Barbro Wadensten, Ulrika Pöder, Inger Jansson, Lena Nordgren

**Affiliations:** 1 Department of Public Health and Caring Sciences, Caring Science, Uppsala University, Box 564, 751 22 Uppsala, Sweden; 2 Centre for Clinical Research Sörmland, Uppsala University, Kungsgatan 41, 631 88 Eskilstuna, Sweden; 3 Institute of Health and Caring Sciences, University of Gothenburg, Box 457, 405 30 Gothenburg, Sweden; 4 Department of Patient safety, Mälar Hospital, 631 88 Eskilstuna, Sweden

**Keywords:** Action research, Critical care, Critical pathways, Grounded theory, Health service research, Implementation, Intensive care, Interprofessional collaboration, Standardized care plan

## Abstract

**Background:**

Clinical pathways can enhance care quality, promote patient safety and optimize resource utilization. However, they are infrequently utilized in intensive care. This study aimed to explain the implementation process of a clinical pathway based on a bottom-up approach in an intensive care context.

**Methods:**

The setting was an 11-bed general intensive care unit in Sweden. An action research project was conducted to implement a clinical pathway for patients on mechanical ventilation. The project was managed by a local interprofessional core group and was externally facilitated by two researchers. Grounded theory was used by the researchers to explain the implementation process. The sampling in the study was purposeful and theoretical and included registered nurses (n31), assistant nurses (n26), anesthesiologists (n11), a physiotherapist (n1), first- and second-line managers (n2), and health records from patients on mechanical ventilation (n136). Data were collected from 2011 to 2016 through questionnaires, repeated focus groups, individual interviews, logbooks/field notes and health records. Constant comparative analysis was conducted, including both qualitative data and descriptive statistics from the quantitative data.

**Results:**

A conceptual model of the clinical pathway implementation process emerged, and a central phenomenon, which was conceptualized as ‘Struggling for a feasible tool,’ was the core category that linked all categories. The phenomenon evolved from the ‘Triggers’ (‘Perceiving suboptimal practice’ and ‘Receiving external inspiration and support’), pervaded the ‘Implementation process’ (‘Contextual circumstances,’ ‘Processual circumstances’ and ‘Negotiating to achieve progress’), and led to the process ‘Output’ (‘Varying utilization’ and ‘Improvements in understanding and practice’). The categories included both facilitating and impeding factors that made the implementation process tentative and prolonged but also educational.

**Conclusions:**

The findings provide a novel understanding of a bottom-up implementation of a clinical pathway in an intensive care context. Despite resonating well with existing implementation frameworks/theories, the conceptual model further illuminates the complex interaction between different circumstances and negotiations and how this interplay has consequences for the implementation process and output. The findings advocate a bottom-up approach but also emphasize the need for strategic priority, interprofessional participation, skilled facilitators and further collaboration.

**Electronic supplementary material:**

The online version of this article (10.1186/s12913-018-3629-1) contains supplementary material, which is available to authorized users.

## Background

Clinical pathways, which are also known as care/critical pathways and by several other terms, form a protocol-based care methodology that has the potential to ameliorate the implementation of current evidence in local practice, enhancing staff knowledge and supporting interprofessional teamwork and communication. Pathway methodology can thereby enhance care quality, promote patient safety, improve patient outcomes, and optimize resource utilization [1–5]. However, contextual circumstances must be taken into account, and general conclusions about pathways should be made with caution [1, 6].

The context of intensive care units (ICUs) is complex, as it includes critically ill patients and their relatives, sophisticated equipment, rapid advancement within medical and nursing care, and a large number of staff with different competences and attitudes who operate in teams with fluid membership [7–9]. The use of pathways in ICUs seems promising, as it can contribute to increased adherence to best-practice guidelines, streamlined care, a decreased mechanical ventilation time, and reduced complications [10–14]. As pathways are still rarely utilized in ICUs, further implementation has the potential to benefit both patients and healthcare providers [15, 16].

The implementation of a pathway typically includes creating/adapting a pathway that is designed for the specific patient group and setting. This creation/adaption includes considering the current situation, essential evidence-based key interventions, local contextual circumstances, interprofessional teamwork, and eventual patient involvement. The implementation further includes activities to ensure that the pathway is used in practice, and evaluation and continuous follow-up to ensure sustainability [17]. Pathways are thus complex interventions, and implementation might be challenging [18, 19]. Existing implementation frameworks/theories, for example the in healthcare studies commonly applied framework ‘Promoting Action on Research Implementation in Health Service’ (PARIHS) [20] and the ‘Consolidated Framework for Implementation Research’ (CFIR) [18], propose that facilitators and barriers for implementation can be related to the characteristics of the innovation/intervention object, recipients/individuals involved, context/setting, and facilitation/process of implementation. This means, for example, that successful implementation is promoted by a learning culture and an object that fits the local setting, has a profound evidence base and entails observable results. Barriers for implementation include resistance to change and/or an object that is not perceived to have a relative advantage for the recipients [18, 20]. Existing research exploring pathways seldom illuminates the implementation process in depth [1, 5, 6, 21, 22]. However, when drawing from lessons from the literature on other types of protocols, there are indications that successful pathway implementation often depends on the engagement of local clinical staff [1, 22].

In Sweden, pathways are in use in only 20% of ICUs [15]. Key persons from those ICUs have witnessed a positive impact of the pathways, such as a better care structure, better quality control and easier documentation. They have also pointed out that the implementation process is complicated and is affect by multiple factors, such as inspiration sources, the project group constellation, available resources, pathway characteristics, implementation activities, and staff characteristics [23]. The study that revealed these findings was, however, retrospective and primarily included the people responsible for pathway implementation, which, in some cases, had occurred several years ago [23]. Although the key informants’ perspectives provided valuable insights to facilitate enhanced pathway use and further understand the implementation process, there was a need for more knowledge. To gain in-depth insights, there was a need to explore an implementation process in real time and include perspectives from different staff categories and managers.

The present study aimed to explain the process of implementation of a clinical pathway based on a bottom-up approach in an ICU context.

## Methods

The study design was emergent. An action research project was carried out in collaboration between a local ICU and the research team [24]. As further described below, a core project group in the ICU managed a clinical pathway implementation with some facilitation from the researchers, and the researchers explored and followed up the process guided by grounded theory methodology [25].

### Setting

The setting was an 11-bed general ICU in Sweden that had the capacity to care for three patients on mechanical ventilation. The ICU staff included intensive care/anesthetist registered nurses, assistant nurses, anesthesiologists, and a part-time physiotherapist. A first-line manager (registered nurse) was the head of the nursing staff (registered and assistant nurses) and was responsible for organizing and staffing the ICU, while an appointed anesthesiologist had medical management responsibilities. A second-line manager (anesthesiologist) managed the first-line manager and the anesthesiologists and was responsible for the Anesthesia Department in which the ICU was located. The physiotherapist had a separate manager who was the head of all the physiotherapists at the hospital. The nursing staff members were primarily stationed in the ICU, while the anesthesiologists also manned the operating theatre, and the physiotherapist also manned other wards. Physicians from the patients’ primary clinic (e.g., cardiologists or surgeons) were involved in medical care but did not participate in daily ICU practice. Other professionals (e.g., occupational therapists, dieticians or counselors) were available as consultants.

Throughout the paper, *staff* refers to all categories of ICU staff. When a particular staff category is referenced, it is specified.

### The action research project

The origin of the action research project was that staff and managers in the ICU perceived some issues in their practice. The issues included unequal care, documentation deficits and role vagueness (further illuminated in the Results section). To overcome these issues, they intended to implement a clinical pathway methodology and contacted the researchers for support. To empower the local ICU, learn from the process and produce understanding and knowledge transferable to others, an action research project was performed. Permission was obtained from the Regional Ethical Review Board (2012/166) and the ICU management.

The project involved staff and managers in the ICU and was managed by a local voluntary core group. The core group included staff members of different professions, all of whom had a wealth of experience in the ICU (three registered nurses, one assistant nurse, one anesthesiologist, and one physiotherapist). To support the core group, two members of the research team were engaged in the project as external facilitators (one doctoral student experienced in intensive care and pathway implementation [PBS] and one senior researcher with extensive experience in qualitative research [LN], both registered nurses).

The project developed and implemented a clinical pathway for the care of patients on mechanical ventilation and evaluated the implementation. The choice of patient group was motivated by the complexity and rapid development of treatment for these patients. Additionally, as mechanical ventilation is a common intervention in ICUs, the staff was supposed to have the opportunity to quickly become familiar with the pathway methodology. The completed pathway included common concerns, goals and care activities for essential matters such as respiration and circulation; communication; knowledge and cognition; nutrition; elimination; skin and tissue; activity; sleep; pain; and psychosocial, spiritual and cultural needs. The associated knowledge base included 18 new/revised local guidelines with references to available evidence.

The project progressed through four emerging partly overlapping phases (see Table 1). Each phase included cycles of ‘observing,’ ‘reflecting,’ planning’ and ‘acting,’ which are significant for action research [24]. For instance, each guideline development step employed at least two cycles. In the first cycle, information was collected and considered (observing and reflecting), priorities were set (planning), and a guideline draft was created (acting). In the second cycle, opinions about the draft were collected and considered (observing and reflecting), implementation or, where applicable, revisions were planned (planning), and the guideline was published or revised (acting). When there were revisions, a third cycle was started, and so on. ‘Acting’ included both development (i.e., activities to develop the pathway and guidelines) and implementation activities (that is, facilitating activities and actually implementing activities). The core project group members were responsible for the content and progress of the project and were the ones who performed the activities in the action research cycles. The external facilitators contributed their experiences, promoted reflection and were primarily responsible for data collection and analysis to explore the process.

**Table 1:** Overview of the action research project, including timeframes for the different phases, the primary content of the different parts of the action research cycles in each phase, and data sources utilized when exploring the process

Project Phase	Time	Content of the Action Research Cycles^a^				Data Source^b^
		Observing and reflecting	Planning	Acting		
				Developing activities	Implementing activities	
1. Initiating and defining the improvement work	November 2011–March 2012	Problem identified inter alia by review of health records. Need for change acknowledged.	Initial planning for a clinical pathway project. Researcher contact established.	Core project group and external facilitators assigned. Patient group chosen for the first pathway. Necessary permits obtained.	Project information provided in nursing and anesthesiologist staff meetings and by e-mail.	Logbooks. Field notes. One FG with the core project group.
2. Exploring and initial drafting	March–November 2012	Current practice and existing guidelines scrutinized and reflected upon.	Planning for developing the pathway and for the need to create new or revise existing guidelines.	External searches and reviews of pathways in other ICUs. Evidence search with librarian assistance. Initial drafting of a pathway and some guidelines.	Pathway methodology lectures (with only nursing staff attending). Involving specialized staff to find evidence for guidelines. Involving staff outside the core group in reviewing drafts.	Logbooks. Field notes. Two core group FGs. One physiotherapist and two manager interviews. Six staff FGs.
3. Revising, completing and implementing	November 2012–October 2014	Perspectives from staff and managers on the drafts were collected and considered.	Perspectives were prioritized, and plans were constructed for revised models of the guidelines and pathway.	Revision and successive intranet publication of guidelines. Composition, clinical testing, and intranet publication of the pathway.	Repeated information in nursing staff meetings. Each guideline e-mailed to all staff. Strategically placed reading copies and sign-up lists. An external lecturer discussed change in sedation regime (reaching all staff categories).	Logbooks. Field notes. Two core group FGs.
4. Enforcing and evaluating	October 2014–September 2016	Use and perceptions of the pathway were evaluated, and the results were reviewed.	Planning for enhanced utilization.		Repeated information and reminders to staff. Feedback from data analysis to managers and staff.	Field notes. Two manager interviews. Four core group interviews. Five staff FGs. Questionnaire. Monthly health record screening.

*Abbreviation*: *FG* Focus group interview. Notes: ^a^ The core project group mainly responsible. ^b^ The external facilitators mainly responsible

### Participants and data collection

The exploration of the clinical pathway implementation was performed with Grounded theory methodology. Guided by a simultaneously performed analysis, the sampling was thus purposeful and theoretical [25]. To promote reflection upon and motivation for the action research project and to obtain a sample with maximum variation, the core project group, all other ICU staff and the first- and second-line managers were informed about the project and the study in staff meetings and by e-mails. Along with this information, they were also invited to participate in the study. In total, 71 staff members/managers participated in the study and contributed data in one or more data sets (see Table 2).

**Table 2:** Frequencies and distributions for staff, managers, and study participants: A. Staff and managers in the intensive care unit (female/male gender distribution within parentheses). B. Study participants by data set (female/male gender distribution within parentheses). C. Summary of study participants by occupation (female/male gender distribution and median values within parentheses)

	Registered nurses	Assistant nurses	Anesthesi-ologists	Physio-therapists	First- and second-line managers	Total
A. Staff and managers in the ICU						
Project start	28 (26/2)	26 (26/−)	6 (2/4)	1 (1/−)	2 (1/1)	63 (57/6)
One-year follow-up	23 (20/3)	27 (25/2)	12 (6/6)	1 (1/−)	2 (1/1)	65 (53/12)^a^
B. Study participants by data set						
Project phases 1–4:						
Core project group – logbooks, focus groups and individual interviews	4 (4/−)^b^	1 (1/−)	1 (−/1)	1 (1/−)		7 (6/1)
Project phase 2:						
Managers – individual interviews					2 (1/1)	2 (1/1)
Staff – focus group interviews	16 (14/2)	13 (13/−)	4 (1/3)			33 (28/5)
Project phase 4:						
Managers – individual interviews					2 (1/1)	2 (1/1)
Staff – focus group interviews	12 (10/2)	7 (7/−)	6 (2/4)			25 (19/6)
Staff – questionnaire	20 (17/3)	18 (16/2)	6 (3/3)			44 (36/8)
C. Study participants by occupation	31 (27/4)	26 (24/2)	11 (5/6)	1 (1/−)	2 (1/1)	71 (58/13)^c^
Age, years (median)^d^	29–64 (52)	24–64 (46)	27–66 (52)			27–66 (51)
Years in occupation (median)^d^	2–40 (24)	1–40 (20)	1–38 (22)			1–42 (23)
Years in the intensive care unit (median)^d^	1–37 (12)	0.5–37 (10)	1–27 (3)			0.5–41 (12)

^a^36 (55%) persons were the same as at the beginning of the project

^b^One of the initial three registered nurses went on leave in project phase two and was replaced by another registered nurse

^c^Each participant contributed to one or more data sets; in total, 77% of the available staff members/managers participated

^d^The physiotherapist and managers had an age range of 50–65 years, with 30–40 years in their occupation and more than 15 years in the intensive care unit. Data were not specified further to maintain individuals’ confidentiality

The data were collected through multiple sources (see Table 1). The core project group kept logbooks, and the external facilitators continuously recorded field notes during meetings/other contacts and based on the documents that were collected from the ICU (drafts, guidelines and the pathway). The logbooks/field notes included activity calendars and reflections [24].

Focus group interviews [26] that were moderated by the external facilitators were performed with the core group and among other staff (1–1.5 h/session). The staff sessions occurred with one staff category at a time to promote an atmosphere that allowed the participants (up to 7/session) to be free to speak. Individual interviews [27] were performed with managers and members of the core group (0.5–1 h/session, interviewer PBS). Written and verbal informed consent was provided by all participants, and confidentiality in the focus group interviews was discussed in each session. To understand the concerns that emerged during the project and in participant interactions, probes were used after the initial question in both focus groups and individual interviews. The areas that were covered in the sessions included current practices and collaboration, the project (initiative, roles, progress and facilitating/hindering factors), the clinical pathway, implementation, and the impact on practice and collaboration. Subsequent sessions were planned based on the analysis of the former. Additional file 1 provides an overview of the semistructured interview guides used. All sessions were recorded and transcribed verbatim.

Questionnaires were distributed to all staff when the pathway had been implemented for approximately 1 year. The composition of the questionnaire was based on a previously used questionnaire [28] with additional questions inspired by previous studies [15, 23, 29], and it encompassed the following: the general impression of the pathway, utilization, patient/family involvement, usability, documentation, care quality, and the implementation process. Additional file 2 provides the questions included in the questionnaire. In addition, two ICU nurses across a two-year period retrospectively screened all health records for the patients who had been on mechanical ventilation (n136), and data on documented pathway use were collected.

### Analysis

Simultaneously, with the data collection, a constant comparative analysis was performed [25]. To inductively identify concepts in the logbooks, field notes and interviews, open coding was performed. The concepts were compared and successively categorized and recategorized. To identify the process and the categories’ interrelations, axial coding was employed. During the selective coding process, a core category was gradually identified, and other categories that emerged were refined and integrated into a preliminary theoretical scheme. To generate descriptive statistics, data from questionnaires and health records were processed using Microsoft Excel 2007 software. These statistical findings were interpreted and integrated with the qualitative data in the constant comparative analysis.

The authors who conducted the data collection performed the initial analysis together. To enhance the emerging understanding of the data, memo writing, diagramming and reflective discussions were performed with the other authors throughout all phases of the analysis. The preliminary theoretical scheme and related narratives were successively reviewed for internal consistency. Flawed categories were saturated through theoretical sampling both by performing additional data collection and by returning to previously collected data. Finally, a conceptual model emerged and was validated with the original empiric raw data.

### Rigor

To establish trustworthiness, credibility, confirmability and transferability were considered as suggested by Lincon and Guba [30]. Credibility was enhanced through data triangulation, inclusion of different staff categories and managers, and nearly 5 years of engagement, enabling an in-depth understanding of the clinical pathway implementation process from several perspectives. Memo writing, peer debriefing and collaboration in the research team, and member checks with the project’s local core team enabled reflectivity. An audit trail and in-depth methodological descriptions enhanced confirmability. Transferability was enhanced through a detailed description of the context and the performed action research project.

## Results

**Fig. 1:**
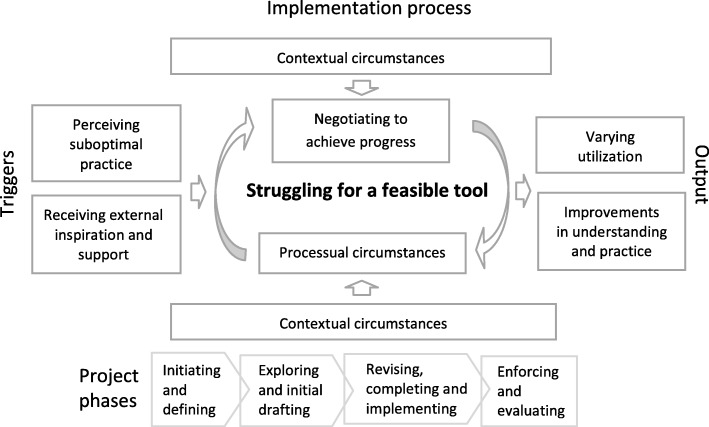
Conceptual model of the process of implementing a clinical pathway based on a bottom-up approach in an intensive care unit

Based on the integrated data, a conceptual model emerged. The model explains the process of implementation of a clinical pathway based on a bottom-up approach in an ICU context. During the implementation process, participants’ primary concern was to make the pathway usable and acceptable in daily practice. The implementation process became tentative and prolonged because it included both facilitating and impeding factors. As some members of the core project group expressed: ‘*There’s been a lot of back and forth*’ and ‘*It’s like you have to walk the mines yourself.*’ Hence, the endeavor to implement a feasible tool became a struggle. A central phenomenon, conceptualized as ‘struggling for a feasible tool,’ emerged as the core category linking all categories. The phenomenon evolved from the ‘triggers,’ pervaded the ‘implementation process,’ and led to the process ‘output’ (see Fig. 1). As illuminated under each heading, all categories in the conceptual model included several subcategories. The comprehensive findings are based on all the different data sources outlined in the Methods section. The narrative includes some quotes from the participants. Additional file 3 provides further examples of quotes that indicate the categories and included subcategories. Additional file 4 provides detailed findings from the questionnaire.

### Triggers

Two major triggers for the implementation process were identified: ‘perceiving suboptimal practice’ and ‘receiving external inspiration and support.’ As expressed by two members of the core project group: ‘*It sort of popped up from several sources. The assistant nurses were at a conference and heard of the pathway methodology, and thought it was interesting/…/and our first-line manager heard from the top level about the obligation to have planned care documented in the health record, the legal demands.*’ *–* ‘*We have considered clinical pathways for a long time but we didn’t feel we could manage an implementation; it felt too big/…/We didn’t have the knowledge, so we contacted you in order to get some help.*’

#### Perceiving suboptimal practice

Perceiving suboptimal practice was indicated by the participants’ experiences of ‘inequalities,’ ‘documentation deficits’ and ‘role vagueness.’

The perceived inequalities were characterized by an insufficient continuity of care and nonpatient-specific variations that made the care dependent on who among the staff was on duty, especially which anesthesiologist was available. This situation was explained by differing levels of competence and commitment, a lack of long-term team planning, and insufficient local guidelines. The existing guidelines were perceived as difficult to find in the electronic information system (intranet) and as not providing a sufficient understanding of tasks. Additionally, for several care activities, there were no guidelines, and there was no tradition of regularly searching for evidence.

The perceived documentation deficits included few health records that displayed planned care (as required by national law) and nursing documentation that was too extensive. As a result, documentation was time consuming, and critical information was difficult to find in the health records. Uncertainties and unawareness about responsibilities and the performance of some tasks, within and between different staff categories, implied role vagueness. The different actors’ partly vague roles meant that collaboration was sometimes perceived as suboptimal, which led to frustration and insecurity. Hence, staff and managers identified a need to improve daily practice.

#### Receiving external inspiration and support

Receiving external inspiration and support included ‘realizing a possible solution’ and receiving ‘contact with external facilitators.’

Staff and managers realized a possible solution for their above-described issues when some nursing staff heard about clinical pathways from other ICUs and from a national conference. However, they did not perceive that they had sufficient competence to conduct an implementation project on their own. When the ICU participated in a national survey on pathway use, some nursing staff saw the opportunity to obtain support for implementing the pathway methodology. The idea was authorized by the first- and second-line managers, contact with external facilitators was initiated, and a project was started.

### The implementation process

The implementation process meant ‘negotiating to achieve progress.’ The negotiations were imposed and affected by both the pre-existing ‘contextual circumstances’ and the emerging ‘processual circumstances’ in a progressing spiral. This dynamic interplay both facilitated and impeded the implementation process and had consequences for the process output.

#### Contextual circumstances

The contextual circumstances originated from both local and general conditions that were related to ‘organization and workplace culture,’ ‘different preunderstanding’ and ‘shifting premises with limited resources.’

The organization and workplace culture in the setting was characterized by an open atmosphere, with social interactions regardless of staff category, and yet a traditional hierarchical structure with the anesthesiologists at the top. As expressed by two anesthesiologists in a staff focus group interview, ‘*It’s very much a team effort/.../Actually, all staff categories are equally important.’ – ‘But, of course, there is some kind of chain of command, so to speak.’*

Interprofessional teamwork was emphasized by all staff categories. Separate managers, staff meetings and guidelines for nursing staff and anesthesiologists, however, compromised common information and reflections. The hierarchical structure meant that although all staff categories could express their views, final decisions were largely those of the anesthesiologists. The organization implied that the project management responsibility was on the first-line manager: As the first-line manager did not have authority over the anesthesiologist, the interprofessional approach in the implementation was complicated. However, the willingness to change was perceived as high overall, which, together with the open atmosphere, facilitated implementation.

The differences in preunderstanding originated from the actors’ different education levels and differing knowledge about evidence utilization and pathway methodology. These differences impeded a shared vision about the clinical pathway and challenged a content that would be suitable for all staff categories. The pathway modeling process thereby became tentative and prolonged. Knowledge deficits among the staff and unfamiliarity with engaging in reading and reflection as a duty led to initial questioning of the project. The core project group thus sometimes felt indebtedness for “just sitting” while their colleagues handled patient care. Shifting premises with limited resources meant that the staff continually had to adapt and juggle competing priorities. The rapid development within intensive care and general decisions within the county council made the staff accustomed to frequent alteration. In contrast, the alterations also meant competing interests and information overload. The following was expressed in a staff focus group interview: ‘*Then, it’s also different circumstances/.../ There is a lot of change here’* (registered nurse).

Periodic staff shortness and fluctuations in care burden meant that the core project group also had to fulfill their ordinary tasks. In addition, a postponed change in the electronic health record system overturned the intended inclusion of the clinical pathway in the system. The pathway and project activities thereby had to be adapted to existing premises. In addition, immediately after the pathway was completed, there were unusually few patients on mechanical ventilation. The decreased number of patients on mechanical ventilation led to limited opportunities to use the pathway, which impeded the implementation. However, the lower frequency of mechanical ventilation was perceived to accentuate the need of a pathway to safeguard quality of care for this patient group.

#### Processual circumstances

The contextual circumstances and negotiations that occurred during the project over time led to processual circumstances. The emerging processual circumstances included ‘vague leadership,’ ‘few interprofessional meetings,’ ‘diffuse vision,’ ‘unequal information and staff involvement,’ ‘delayed follow-up’ and ‘enthusiasm, support and facilitation.’

The vague leadership was characterized by unclear roles and responsibilities in the core project group, managers who assumed a primarily passive role because they relied on the core group’s ability to interpedently operate the project, and external facilitators who sought to promote local ownership of the project and refrain from manipulating the process. As expressed in an interview with one of the managers: ‘*I haven’t interfered/.../ Maybe scheduled check-offs would have been beneficial/.../ We really didn’t know the need, that’s what we understand when looking back.’*

The above situation emerged from the organization and workplace culture and positions in the role setting, as well as working methods. Overall, vague leadership affected all domains of the negotiation and contributed to a tentative and prolonged implementation process. The tentative and prolonged process was perceived as bothersome but educational and empowering, and it enabled staff to become used to the idea before the pathway was implemented.

The organization and workplace culture that separated nursing and medical staff and the positions in the role setting and working methods implied few interprofessional meetings. This situation, combined with varied levels of preunderstanding and vague leadership, led to a diffuse vision. The diffuse vision originated in conflicting ideas about the clinical pathway format, the extent of the knowledge base, evidence base/references, the interprofessional scope, and inclusion in the health record, which impeded the pathway modeling and activity timing.

Altogether, the above circumstances contributed to unequal information and staff involvement. For instance, although information was repeatedly provided in nursing staff meetings, no corresponding information was provided for the anesthesiologists. This situation, combined with high staff turnover and many new employees, led to limited involvement by the anesthesiologists in pathway development, which impeded an interprofessional approach. As indicated both in the focus group interviews and by the questionnaire answers, most nursing staff felt informed and included in the implementation process, while many anesthesiologists did not.

The implementation was also impeded by a delayed follow-up, which emerged from the shifting premises, the compromises in activity timing, and the vague leadership. For instance, when the pathway was completed, several of the project’s core group members went on to other assignments and did not have the time or commitment to follow-up on the implementation. Initially, no one inherited the mission, which led to a decreased focus on the pathway. When the role was eventually filled, follow-up was conducted and feedback provided, which was perceived to increase awareness of the pathway and facilitate utilization.

The processual circumstances explained above largely impeded the implementation process. The process was, however, facilitated and enabled by the enthusiasm and support that emerged from the workplace culture and implementation activities. The enthusiasm and support included trust in the advantage of the tool and internal support among the nursing staff in the core project group, as well as emotional support from the first-line manager. Taken together, these contributors prevented the core group from giving up in times of despair. The enthusiasm and support also included the internal facilitation that the core group provided to the staff. Based on both interview and questionnaire findings, the internal facilitation was essential. Important facilitation strategies included information, involvement encouragement, practical support, training and reminders. Together with the willingness to change among nursing staff, these efforts promoted implementation. The action research project design and involvement of external facilitators were perceived to enhance the pathway’s legitimacy. The following was expressed by a member of the core project group: ‘*To be able to say, Well, I have to leave at one o’clock because I have a meeting with XX and XX* [the anesthesiologist and the physiotherapist in the core project group] *and some researcher, gives much more weight to my job /…/ suddenly, this is something really serious that we do.’*

The core project group and managers perceived support from the external facilitators in the implementation process. Participation in data collection and feedback on the results promoted reflection in the core group, as well as by the managers and among other staff members, and this reflection was perceived to facilitate implementation.

#### Negotiating to achieve progress

The actors addressed the circumstances by negotiating to achieve progress, which implied flexibility and pragmatism, as well as orientation-reorientation, with respect to both the actors’ own preunderstanding and to interactions with others. The negotiations occurred in ‘role setting and working methods,’ ‘pathway modeling’ and ‘activity timing.’ As expressed by members in the core project group: *‘It’s difficult to have time allocated, an hour here and there. It’s not enough to get into work’* (logbook data). ‘*We had a vision in the beginning about an interprofessional approach, but we later understood that it wouldn’t be possible.’ – ‘Yes, there’s been a lot of back and forth’* (conversation in a focus group)*.*

The negotiations in role setting and working methods included the core project group’s activities, managers’ and external facilitators’ commitment, and different staff categories’ priorities and engagement. For instance, having an interprofessional core group and cooperation with researchers/external facilitators was perceived to promote the clinical pathway’s quality and project’s legitimacy. However, the core project group’s nursing members became a working group that developed drafts and only occasionally consulted the other team members. This compromise was imposed by the limited time for the anesthesiologist (due to short staff) and for the physiotherapist (due to part-time availability in the ICU) in the project and the positions reflecting the managers’ and external facilitators’ level of involvement. The solution allowed the project to be completed but also impeded the intention of interprofessional meetings. Furthermore, to utilize existing knowledge and promote acceptance for the pathway, the core group intended to involve other staff in its creation to obtain both evidence and feedback on drafts. Initially, however, there was some resistance to engagement. This resistance was explained by competing interests, information deficits, a high burden of care, and vague leadership. Negotiations of priorities thus occurred, and as the core project group enhanced their support for the involved staff, involvement and willingness to change increased.

The negotiations in the pathway modeling included a trial-and-error process that was imposed by varied preunderstanding and the diffuse vision of the pathway. For instance, the intent was to make the pathway and applicable guidelines interprofessional. However, varied preunderstanding and resistance from anesthesiologists forced the core project group to reorient and develop separate yet consistent guidelines for medical and nursing tasks. The structural decisions within the county council inhibited the intent to include the pathway in electronic health records. Additionally, the pathway’s status was questioned by the administrative staff, which delayed accessibility via the intranet. These multiple orientation-reorientation turns led to a tentative and prolonged process, but finally the negotiations led to a completed clinical pathway.

The negotiations in activity timing were imposed by the shifting premises and affected by vague leadership and the diffuse vision, which led to a prolonged process. For instance, as the project proceeded, there were fewer registered nurses available, which enforced negotiations for time. Allotted project days were scattered, and although some periods with low burdens of care allowed for project activities during spare time, the project was paused during several periods, which made the work ineffective. According to the initial plan, the pathway and all the guidelines that were incorporated in the knowledge base were to be implemented in one bundle within 1 year from the project start, in conjunction with the department’s yearly training days. Because the development took more time than expected, the guidelines were instead successively implemented. When all acquired guidelines were implemented (2.5 years after the project start), the pathway was completed, and a “release party” was arranged, which included practical training and reflections on the pathway use. The delayed accessibility via the intranet, however, meant that the pathway was not taken into use until 4 months later. As expressed in a staff focus group interview: ‘*There was a release party and then came summer. Later, when we were supposed to use it, we had forgotten what they said, sort of’* (assistant nurse).

By the time the clinical pathway was supposed to be used, the motivation from the release party had faded. Because other tasks were prioritized, a planned retraining was never completed. Taken together, these circumstances led to decreased focus on the pathway.

### Output

The output of the implementation process included ‘varying utilization’ of the clinical pathway while perceiving ‘improvements in understanding and practice.’

#### Varying utilization

Varying utilization included the clinical pathway being primarily utilized as a ‘nursing tool’ and ‘fluctuating documentation’ of pathway use.

The pathway as a nursing tool emerged as the registered nurses became the primary users, which was prominent in both interviews and questionnaire findings. The pathway was intended for interprofessional use, but as expressed by an experienced assistant nurse: ‘*It’s more like it is the registered nurses’ administrative task in some way’* (data from staff focus group).

Newly employed assistant nurses perceived that the pathway was supportive, but the experienced assistant nurses often performed tasks without referencing the pathway. Several anesthesiologists expressed that they did not use the pathway because they relied on nursing staff to handle patient care. Despite these circumstances, most staff, regardless of profession, based on interview and questionnaire findings, perceived the pathway as useful and the implementation as successful. In the questionnaire they stated that the pathway was frequently utilized. However, although patient autonomy was desired, the decision to use the pathway was often made without involving or informing the patients or their families.

Fluctuating documentation was characterized by a varying number of health records from patients who had been on mechanical ventilation including documentation on pathway use. In some months, there were no health records that included pathway documentation, while in other months, all records included pathway documentation. The proportion increased for some months after the health record screening started and when feedback on results had been provided but did not appear to be affected by the focus groups or the total number of patients on mechanical ventilation. However, documentation appeared to be related to the length of time on mechanical ventilation. Just under a tenth of the health records from patients who had been on mechanical ventilation for less than 1 day (n66) included pathway documentation, while approximately half of the patients who had been on mechanical ventilation for 1 day or more (n70) had records that included pathway documentation. The maximum proportion of records that included pathway documentation (approximately three quarters) was from the fourteen patients who had been on mechanical ventilation for approximately 1 week.

#### Improvements in understanding and practice

Perceiving improvements in understanding and practice was indicated in both interview and questionnaire findings. The core project group, managers and staff perceived the clinical pathway as a feasible tool that facilitated their daily practice and, to some degree, contributed to overcoming the issues that triggered the implementation process. The improvements were perceived to include ‘knowledge expansion,’ ‘clarified roles,’ ‘improved documentation’ and ‘care equality.’ Staff and managers thus perceived enhanced quality of care and patient safety. The following was expressed by one manager: ‘*In fact, it has meant a lot of things, partly because we have worked in a more structured way to further develop the care/.../ We are more certain that we have increased the basic quality level/.../ Before there were a lot more fluctuations, a lot more roller coaster in the care. Now it’s a bit more equal. And you don’t have to write as much because everybody knows we follow the clinical pathway.’*

The knowledge expansion included care for the patient group and general knowledge, as explained by a focus on the care, reflection, and participation in the project. The new/revised guidelines were perceived, especially by the assistant nurses, to promote an understanding of different procedures. General knowledge expansion was characterized by managers and nursing staff who enhanced their methodological understanding of running a project, evidence-based practice, research utilization and methods. This knowledge was perceived as useful for future improvement projects. The members of the core project group additionally perceived personal development, and several proceeded to more advanced duties after the pathway was completed.

The pathway contributed to clarified roles for different staff categories, which to some extent increased interprofessional collaboration, ensured that nothing was forgotten, and provided staff and managers with a sense of security. For instance, previous uncertainties for some tasks related to the endotracheal tube were resolved, which meant closer collaboration between registered and assistant nurses. Nursing staff also referred to the pathway when they suggested actions to anesthesiologists, which enhanced their professional confidence and facilitated acceptance for the suggestions.

The improved documentation included minimized daily text volumes in the health record, which made documentation easier to survey. Additionally, when the pathway was used, the legal requirement for documenting care plans was fulfilled. Information transfer was complicated because the pathway was not included in the electronic health records, but the printed text was perceived as convenient to read. Based on both the interview and the questionnaire findings, the pathway was perceived to guide care, facilitate planning, and make the applicable guidelines easier to find.

Taken together, the above improvements were perceived to facilitate evidence-based practice, enhance continuity of care and decrease nonpatient-specific variations, thus enhancing care equality. Thereby, care was less dependent on the staff members who were on duty. However, although care continuity was perceived as improved, the care process remained largely dependent on the anesthesiologist on duty.

## Discussion

The present findings explain a bottom-up implementation of a clinical pathway in an intensive care context. The generated conceptual model illuminates the complex interplay between different circumstances and forced negotiations that in turn had consequences for the implementation process and output. Since this process, to our knowledge, has not been previously explained, the findings provide a novel understanding that can be useful in future implementation processes.

The conceptual model was generated from empirical findings but resonates well with existing implementation frameworks/theories For example, the in healthcare studies commonly applied PARIHS framework [20] and CFIR [18]. The present findings especially strengthen the recently suggested adjustment of the PARISH framework, emphasizing the importance of the recipients of the innovation/implementation object and the need for facilitation [20].

Based on the empirical findings, the core of the clinical pathway implementation process was conceptualized as ‘struggling for a feasible tool.’ The issues that the staff and managers wanted to solve were care inequalities, documentation deficits and role vagueness. The pathway methodology was chosen because it can amend such issues [1–5]. However, consistent with others’ experiences [23, 31], developing a pathway that was suitable for the local setting and completing the implementation formed a complex mission that required a struggle and more time than expected.

Clinical pathways are complex interventions, and it is difficult to precisely define the active components of their impact [19]. Based on the participants’ perceptions, the pathway that was developed in the ICU was feasible, and the implementation was successful. The participants perceived improvements in understanding and practice, which contributed to reducing, although not completely erasing, issues with inequalities, documentation deficits and role vagueness. The positive perception of the pathway is aligned with findings from several other studies and contradicts conclusions that nursing staff are negative toward pathways due to medical language and that clinicians feel that pathways threaten their clinical autonomy, as revealed in some other studies [21, 22, 31]. The perceived improvements in understanding and practice indicate that the implementation process could be one of the active components of a pathway’s impact on practice, as previously proposed [1, 17]. However, there was varied pathway utilization, which may have delimited the impact. Because the present study did not explore patient outcomes, we cannot draw firm conclusions.

The primary facilitators in the implementation appeared to be local enthusiasm and motivation to improve practice, the core project group serving as internal facilitators, staff involvement, and a first-line manager who supported the initiative. The current findings confirm the strength of engaging clinical staff (the recipients) in the process, as advocated by both implementation researchers [1, 18, 20, 22] and action researchers [24, 32]. The bottom-up approach led to a feeling of local ownership for the project and enabled internal facilitators, training and reminders, which, consistent with previous findings [22, 23, 29], promoted motivation and willingness to change. The local engagement enabled clinical experiences to be incorporated with scientific evidence. According to Andersson et al. [33], this aspect could further explain the general positive perception of the pathway. Additionally, Weinert and Mann [34] propose that combining implementation with a research agenda may be attractive to ICU staff, which was also indicated in the present study. The collaboration with researchers in the action research design was perceived to enhance the project’s legitimacy, and as proposed by Soh et al. [32], the educating approach in the design contributed to improvements in understanding and practice.

The bottom-up approach and the lack of a predefined design also included challenges. Prominent in the findings were the multiple negotiations that occurred throughout the implementation process. The negotiations were imposed by the circumstances and were performed to achieve progress, but in some cases, the compromises impeded implementation. Thereby, as previously proposed [1, 18] and highlighted in the recent refinement of the PARIHS framework [20], it is critical to emphasize the role of the actors who are involved and affected by the implementation and the importance of facilitators.

Staff turnover meant that nearly half of the staff changed during the study period, which impeded involvement and information during the implementation. However, as pathways are especially supportive for new staff [35], the staff turnover accentuated the pathway’s usefulness.

The primary impeding circumstances appeared to be vague leadership and limited interprofessional collaboration, which led to a diffuse vision, varied staff involvement and a delayed follow-up. Although the project was authorized by the ICU management and although the action research design enhanced legitimacy, the project lacked strategic priority. The managers assumed a passive role, and the external facilitators often refrained from manipulating the process. Furthermore, other contemporaneous alterations led to competing interests. Thereby, there was limited devoted time and support, which are impeding circumstances that have also been described in other settings [22, 23, 31].

Facilitation should be operationalized through a network of novice, experienced and expert facilitators to enable the application of skills and strategies to structure the implementation process, manage relationships between the actors, and identify and negotiate barriers [20]. For successful implementation, interprofessional project groups and credible opinion leaders are important [1, 17, 18, 22, 32]. Formally, the current project’s core group was interprofessional, but as in many other improvement initiatives [33], registered nurses were the most represented staff category. Given that these nurses and the participating assistant nurse had great credibility, they were adequate opinion leaders for the nursing staff. However, the anesthesiologists made limited contributions because they did not have an equivalent opinion leader and were not as informed or involved in the process as the nursing staff, which might explain the perception of the pathway as a nursing tool and its limited use among anesthesiologists.

Due to physicians’ authoritative position, their participation is important for successful implementation [17, 36]. However, difficulties involving all staff and conflicts due to diverse professional backgrounds commonly impede implementation [32, 37]. A traditional hierarchical structure, as in the present setting, could compromise interprofessional collaborations [38, 39]. Reeves et al. [40] propose that hierarchical structures are closely related to a culture of animosity about shared professional power. Because the traditional hierarchy has physicians/anesthesiologists at the top, it might be assumed that the responsibility for limited interprofessional interactions rests on this group. However, intensive care registered nurses have a high professional status in Swedish intensive care. Is it thereby possible that they consciously or unconsciously exclude the ‘higher’ profession to shield their area of power? Issues of hierarchy and interprofessional equality merit further exploration.

An additional issue that merits attention is the conflict between available resources and the legal and ethical requirements for healthcare professionals to work according to evidence-based practice and to contribute to improvements in care. To develop more sustainable healthcare organizations, it is essential to engage clinical staff [41], and bottom-up approaches can enhance successful pathway implementation [22, 23]. To develop, implement, evaluate, and maintain pathways or other protocols is, however, time-consuming and places great demands on the local organization. Pathway implementation and evaluation should be continuous processes [17, 22]. In the present study, follow-up was delayed, which impeded implementation, and the participants additionally expressed doubts about their ability to keep the pathway up to date in the future. A lack of systematic pathway evaluation is common [23, 29, 31, 42]. In a recent Swedish national survey, one-third of the reviewed pathways were more than 3 years old, and the quality varied [15]. Thus, there is a need for national collaboration and guidance, indicating that using a predefined structure, such as the ‘7-phase method,’ to design, implement and evaluate pathways [17] could be beneficial. The present findings also further illuminate that inadequate information/electronic health record systems are barriers for pathway implementation [15, 42]. To facilitate increased pathway implementation and enable systematic follow-up and variance analysis, this issue merits greater attention by healthcare managements.

### Clinical implications

Based on the present findings and previous publications, future clinical pathway implementation may be facilitated by embracing a bottom-up approach and advantageously incorporating action research methodology and a predefined structure. The implementation should be strategically prioritized by the management and assigned sufficient resources, with clear leadership and expression of roles and responsibilities. It is important to ensure interprofessional collaboration, to involve all affected staff categories and managers and to include patients’ and relatives’ perspectives. The consequences of imposed negotiations should be considered, and support should be provided by facilitators who have skills and strategies to structure the process, manage the actors’ relationships, and identify and negotiate barriers in the implementation process.

To facilitate enhanced pathway use and enable systematic follow-up, there is a need for national and international guidance and collaboration. Further, hierarchical structures and inadequate information/electronic health record systems must be addressed.

### Methodological considerations

Action research [24] was employed to empower clinical staff, and grounded theory [25] provided rigor to the analysis, promoted theorizing and enhanced the understanding that was obtained via the actions. Hence, as Dick [43] suggests, participants achieved enhanced knowledge and understanding that was usable in the local context, and more general knowledge and understanding were produced, which may be transferable to other contexts.

Limitations included the single setting and that the findings were primarily based on participants’ perceptions, which might not have reflected the complete situation. Because the health record screening revealed limited documentation on pathway use, it is possible that the staff that chose not to participate had negative perceptions that were not captured in the data. Furthermore, no patients or relatives participated in the study. The study focused on the process, so formative evaluation and patient outcomes were beyond the present scope.

To establish trustworthiness, in-depth methodological descriptions were provided, an audit trail was maintained, and data triangulation and peer debriefing were performed. Credibility was enhanced with reflectivity, memo writing, and collaboration with the core project group and research team [24, 30]. The chosen approach implied the researchers’ involvement in the studied process. Balancing active participation and neutrality [44], to facilitate progress while producing valid findings, was a delicate mission. The researchers strived to be open and responsive to and reflective on the core project group’s interests and ideas. However, based on the findings that revealed insufficient leadership as an aggravating factor, the researchers, as external facilitators, could have intervened to a greater extent to expedite the project. Additional involvement, however, might have been perceived as an intrusion on local ownership and, thereby, jeopardized the ambition to study a bottom-up approach of the implementation process, the educational benefits of the project and the sustainability of the implementation.

The quality of grounded theory should be assessed based on the criteria ‘fit,’ ‘work,’ ‘relevance’ and ‘modifiability’ [45]. The present conceptual model emerged from empirical data and fit the context under study. The model works because it explains the implementation process. It is relevant because it reflects a real concern of the people involved. Finally, the model is modifiable because it is open to further development.

## Conclusions

The present findings explain, in a novel way, a bottom-up implementation of a clinical pathway in an intensive care context. The core of the implementation process was conceptualized as ‘struggling for a feasible tool.’ The conceptual model was generated from empirical findings but resonates well with previous findings concerning pathway implementation and consolidated conclusions from current implementation frameworks/theories. The findings illuminate the complex interaction between different circumstances and forced negotiations and how this interplay has consequences for the implementation process and output. A bottom-up approach is advocated, and the need for strategic priority, interprofessional participation, skilled facilitators and further collaboration is emphasized.

Future research on clinical pathways could include patients’ and relatives’ perspectives and evaluate patient outcomes. The field would also benefit from further research on implementation processes that are based on a predefined structure.

## Additional files

Additional file 1: List of topics/questions in the focus groups and individual interviews. This file includes the semi-structured interview guides used in the focus groups and individual interviews (translated from Swedish to English). (PDF 711 kb)

Additional file 2: Content of the questionnaire. This file includes the questions included in the questionnaire answered by staff in project phase four (translated from Swedish to English). (PDF 899 kb)

Additional file 3: Examples of quotes from participants. This file includes some examples of quotes (translated from Swedish to English) that indicate the categories and included subcategories in the conceptual model presented in the results section of this paper. (PDF 724 kb)

Additional file 4: Findings from the questionnaire. This file includes descriptive statistics from the questionnaire answered by staff in project phase four, about 1 year after implementation of the clinical pathway. (PDF 989 kb)

## Abbreviations

AN: Assistant nurse (used only in additional files)
CFIR: Consolidated Framework for Implementation Research
CP: Clinical pathway (used only in additional files)
FG: Focus group interviews (used only in Table 1 and in additional files)
ICU: Intensive care unit
PARIHS: Promoting Action on Research Implementation in Health Service
RN: Registered nurse (used only in additional files)
