# Supplementary material for: Struggling for a feasible tool – the process of implementing a clinical pathway in intensive care: a grounded theory study
Source: BMC Health Serv Res. 2018 Nov 6;18:831. doi: 10.1186/s12913-018-3629-1 (PMC6219016; doi:10.1186/s12913-018-3629-1)
Supplement: Supplementary file 1 — List of topics/questions in the focus groups and individual interviews. This file includes the semi-structured interview guides used in the focus groups and individual interviews (translated from Swedish to English). (PDF 711 kb) [file 12913_2018_3629_MOESM1_ESM.pdf]

Additional File 1: List of topics/questions in the focus groups and individual interviews. All sessions took the form of conversation in which a semi-structured guide was used. The topics/questions were used as a reminder for the interviewer and not rigorously followed. Probe questions, such as: 'Can you give an example?´ or 'What do the rest of you think about that?´ etc. were used after the initial question. Based on how the conversations emerged, the questions were adjusted. Subsequent sessions were planned based on the analysis of the former.

Session	Topics/questions in the semi-structured interview guides
**Focus group 1 with the core project group** (Project phase 1)	• Can you please tell me about how the idea to implement a CP came up? (motive, initiative, expected outcome)<br>• Can you please tell me about the project? (roles, plans, next step, implementation strategies)
**Focus group 2 with the core group** (Project phase 2)	• Can you please tell me about the project? (roles, progress, barriers, support, thoughts, emotions, reactions)<br>• Follow-up last section and researchers' interpretations (expected roles, concerns about commitment and allotted time)<br>• Current plans? (implementation strategies, information/training, follow-up, evaluation)<br>• Expectations? (staff reactions, managers' reactions, roles)
**Individual interviews with managers** (Project phase 2)	• Can you please tell me about the initiative to implement a CP? (motive, initiative, expected outcome)<br>• Can you please tell me about the project? (assignment of core project group, own role, plans, implementation strategies, expectations)
**Focus groups with staff (RNs, ANs, anesthesiologists) and individual interview with the physiotherapist** (Project phase 2)	• Can you please tell me about an ordinary day in the ICU? (tasks, responsibilities, emotions)<br>• How do you care for a patient on mechanical ventilation? (plans, decisions, collaboration)<br>• Who is involved? (different staff categories, patient partnership, relatives)<br>• What do you base the everyday decisions on? (finding information, equality, routines/guidelines)<br>• How do you act when a change is proposed? (change wiliness, learning culture, attitudes)<br>• What are your thoughts on the upcoming CP implementation? (expectations on the project, expected outcome)<br>In the later focus group sessions:<br>• Follow-up on concerns raised regarding diffuse roles and responsibilities<br>• What do you think about your tasks and responsibilities? (appropriate, most important)
**Focus group 3 with the core project group** (Project phase 2)	• Can you please tell me about the project? (roles, progress, barriers, support, thoughts, emotions, reactions)<br>• Follow-up last section and researchers' interpretations (information problems, design of drafts, extension of the knowledge base)<br>• Current plans? (next step, implementation strategies, information, training, follow-up, evaluation)
**Focus groups 4 and 5 with the core group** (Project phase 3)	• Can you please tell me about the project? (progress, barriers, support, thoughts, emotions, reactions)<br>• Follow-up last section and researchers interpretations (extension of the knowledge base, roles)<br>• How do the implemented guidelines work?<br>• Current plans? (next step, implementation strategies, information, training, follow-up, evaluation)
**Individual interviews with core project group members and managers** (Project phase 4)	• Can you please tell me how the project turned out? (implementation successful/unsuccessful, utilization, impact on care, collaboration)<br>• What was your role? (thoughts, emotions, reactions, progress, barriers, support)<br>• Thoughts about sustainability?<br>• If you had a colleague who intended to implement a CP in her/his intensive care unit, what advice would you give?
**Focus groups with staff (RNs, ANs, anesthesiologists)** (Project phase 4)	• Can you please tell me how the project turned out? (reactions, progress, barriers, support, implementation strategies)<br>• What were your roles? (participation, contribution thoughts, emotions)<br>• What do you think about the CP? (impact on care, collaboration, usability)<br>• What do you think about your tasks and responsibilities? (appropriate, most important)<br>• If you are unsure about a task, how do you find information?<br>• Teamwork function, situations when teamwork fails?

Abbreviations: CP, clinical pathway
