# Supplementary material for: Struggling for a feasible tool – the process of implementing a clinical pathway in intensive care: a grounded theory study
Source: BMC Health Serv Res. 2018 Nov 6;18:831. doi: 10.1186/s12913-018-3629-1 (PMC6219016; doi:10.1186/s12913-018-3629-1)
Supplement: Supplementary file 2 — Content of the questionnaire. This file includes the questions included in the questionnaire answered by staff in project phase four (translated from Swedish to English). (PDF 899 kb) [file 12913_2018_3629_MOESM2_ESM.pdf]

Additional File 2: Content of the questionnaire. The original questionnaire was in Swedish and based on previously used questionnaires; see notes.

Questions	Response alternatives
**Background questions** [1]	
Workplace	Free text
Profession	Occupational therapist/ Physiotherapist/ Assistant nurse/ Registered nurse/ Physician/ Other (free space to specify)
Years in profession	Free text
Years at the current workplace	Free text
Employment	Full-time/ Part-time/ By hour<br>Daytime/ Nighttime/ Both
Age	Free text
Gender	Male/ Female
**The implementation process**	
Have you been involved in the process of developing and implementing the clinical pathway? [2]	Yes, by: … (space for free text) / No
What strategies were used when implementing the CP? [1, 3]	
Several alternatives (a–j) can be selected<br>a. Internal facilitators, i.e. local person available for questions<br>b. External facilitators, i.e. person from outside available for questions and educations if needed<br>c. Training prior to implementation<br>d. Ongoing training<br>e. Reminders after implementation<br>f. Printed matter<br>g. Computerized support<br>h. Feedback and evaluation on one occasion<br>i. Regular feedback	**a–i:** Yes/ No/ Do not know
j. Other	Please specify:… (space for free text)
Did you perceive the implementation of the CP to be successful? [1, 3]	Yes/ No (and space for free text comment)
Was there anything that especially facilitated the implementation of the CP? [1]	Yes/ No (and space for free text)
What strategies (a–i) were important to achieve successful implementation of the CP? [2]<br>a. Internal facilitators<br>b. External facilitators<br>c. Training prior to implementation<br>d. Ongoing training<br>e. Reminders after implementation<br>f. Printed matter<br>g. Computerized support<br>h. Feedback and evaluation on one occasion<br>i. Regular feedback	**a–i:** Totally agree/ Partly agree/ Partly disagree/ Totally disagree/ Not used
Which categories of staff utilize the CP? [1] Several alternatives can be selected.	Registered nurses/ Assistant nurses/ Anesthesiologists/ Physiotherapist/ Physicians from other clinics/ Other (please specify: …) Comment… (space for free text)
When you care for a patient with mechanical ventilation, how often do you perceive the CP is utilized? [1]	Always/ Often/ Seldom/ Never (and space for free text comment)
In what way are the patients involved in the decision to use the CP (when their condition allows)? [1]	Participate in decision/ Informed that CP is used/ Neither/ Do not know (and space for free text comment)
In what way is family/next of kin involved in the decision to use the CP? [1]	Participate in decision/ Informed that CP is used/ Neither/ Do not know (and space for free text comment)
How do you act when your patient has needs not covered by the CP? [1]	Commonly we:<br>End the CP and make up an individual care plan/ End the CP and continue without a written plan/ Supplement the CP with an individual plan/ Continue to use the CP and adjust the care, without any new written plan/ The patient receives the care stated in the CP, without adjustment to individual needs (and space for free text comment)
Any views you want to add? [1]	Free text space

Notes: See next page

Continued on next page

Continued from previous page

**Perceptions of working with the clinical pathway** [4]	
**Usability**	
I feel that it is easy to work according to the CP	Totally agree/ Partly agree/ Partly disagree/Totally disagree
It is easier and quicker to read and get knowledge about a patient who is cared for according to the CP than a patient without the CP	Totally agree/ Partly agree/ Partly disagree/Totally disagree
It is easier to see which nursing measures have been taken when the patient has the CP than without the CP	Totally agree/ Partly agree/ Partly disagree/Totally disagree
The use of the CP helps me structure my work so that nothing gets forgotten	Totally agree/ Partly agree/ Partly disagree/Totally disagree
I feel that it is easy to work with the CP on our ward	Totally agree/ Partly agree/ Partly disagree/Totally disagree
**Documentation**	
I feel that it is quicker to document when the CP is used than without the CP	Totally agree/ Partly agree/ Partly disagree/Totally disagree
I feel it is easy to document when the CP is used	Totally agree/ Partly agree/ Partly disagree/Totally disagree
I feel that double documentation has decreased with the use of the CP	Totally agree/ Partly agree/ Partly disagree/Totally disagree
It is easier to see what needs to be done when the patient has the CP than if documentation consists of running notes	Totally agree/ Partly agree/ Partly disagree/Totally disagree
My methods of documentation become restricted when I work according to the CP	Totally agree/ Partly agree/ Partly disagree/Totally disagree
**Quality of care**	
I feel that I have had more time for patient-focused care once the CP was implemented	Totally agree/ Partly agree/ Partly disagree/Totally disagree
I get a quick overall view about the patient's condition when using the CP	Totally agree/ Partly agree/ Partly disagree/Totally disagree
I feel that patients cared for according to the CP receive good and safe care to a greater extent	Totally agree/ Partly agree/ Partly disagree/Totally disagree
The knowledge overview related to the CP has been helpful for me to understand why we should care for the patient in accordance with the measures in the CP	Totally agree/ Partly agree/ Partly disagree/Totally disagree
I do not deviate from the CP unless the patient's condition necessitates an individual care plan	Totally agree/ Partly agree/ Partly disagree/Totally disagree
I have greater trust in my own work experience than in the content of the CP	Totally agree/ Partly agree/ Partly disagree/Totally disagree
It is easier for new staff to care for a patient with the CP than without the CP	Totally agree/ Partly agree/ Partly disagree/Totally disagree
The use of the CP facilitates the introduction of new staff	Totally agree/ Partly agree/ Partly disagree/Totally disagree
I was informed that the knowledge overview related to the CP is supposed to be based on evidence	Totally agree/ Partly agree/ Partly disagree/Totally disagree
**Implementation process**	
I was well aware of what a CP was when it was time to start using it	Totally agree/ Partly agree/ Partly disagree/Totally disagree
I continuously received information about the work to develop the CP	Totally agree/ Partly agree/ Partly disagree/Totally disagree
I felt well informed about the expected benefits of using the CP	Totally agree/ Partly agree/ Partly disagree/Totally disagree
I felt that I participated in the work to develop the CP	Totally agree/ Partly agree/ Partly disagree/Totally disagree
**General impression**	
What is you general impression of the CP?	**Positive** It facilitates my work/ **Negative** It hinders my work

Abbreviation: CP: Clinical pathway.

Notes: [1)] Based on questionnaires used in previous studies (Bjurling-Sjöberg, Jansson, Wadensten, Engström, & Pöder, 2014 [15]; Bjurling-Sjöberg, Wadensten, Pöder, Nordgren, & Jansson, 2015 [23]). [2)] Study-specific question. [3)] Based on questionnaire used in previous study (Jansson, Bahtsevani, Pilhammar-Andersson, & Forsberg, 2010 [29]). [4)] Questions based on a questionnaire by Jakobsson and Wann-Hansson, 2013 [28]. For full references, see main paper.
