# Supplementary material for: Struggling for a feasible tool – the process of implementing a clinical pathway in intensive care: a grounded theory study
Source: BMC Health Serv Res. 2018 Nov 6;18:831. doi: 10.1186/s12913-018-3629-1 (PMC6219016; doi:10.1186/s12913-018-3629-1)
Supplement: Supplementary file 3 — Examples of quotes from participants. This file includes some examples of quotes (translated from Swedish to English) that indicate the categories and included subcategories in the conceptual model presented in the results section of this paper. (PDF 724 kb) [file 12913_2018_3629_MOESM3_ESM.pdf]

Additional File 3: Examples of quotes from participants. Some examples of quotes that indicate the categories, and included subcategories, in the conceptual model that explains the process of implementing a clinical pathway based on a bottom-up approach in an intensive care unit context.

Core category: Struggling for a feasible tool

**Categories** (included subcategories)	**Examples of quotes (translated from Swedish)***
**Triggers**	
**Perceiving suboptimal practice** (Inequalities, Documentation deficits, Role vagueness)	- It's very dependent, as said, on which anesthesiologist is on duty in the ICU, if it's someone who pushes the care forward or if it's someone who just let it float on (RN).<br>- How much has this helped the patient, jumping from one anesthesiologist's decision to other anesthesiologist's decision (manager)?<br>- Before the CP, it was a bit like: this I usually do and it's usually good. In fact, that was what we wanted to overcome (RN).<br>- But it's very clear in this document [how to control ventricle retention]. – No, I do not think it's clear, it doesn´t explain why (two ANs).<br>- Unfortunately, there is no policy to evidence base, and no time for searching evidence (member of core project group).<br>- Over the last years we have tried to achieve better documentation/…/ we had difficulties prioritizing (member of core project group).
**Receiving external inspiration and support** (Realizing a possible solution, Contact with external facilitators)	- It sort of popped up from several sources. The ANs were at a conference and heard of the CP methodology, and thought it was interesting/…/ And our first-line manager heard from the top level about the obligation to have planned care documented in the health record, the legal demands. – We have considered CPs for a long time but we didn't feel we could manage an implementation; it felt too big/…/We didn't have the knowledge, so we contacted you in order to get some help (two core group members).<br>- Well, I had the goal for a long time that new staff should be able to read information and that it should be easy for them to find. But I didn't really know how to achieve this/…/ How to take the step over to DOING/…/ And when we had the opportunity to get support, it was liberating (manager).
**Implementation process**	
**Contextual circumstances** (Organization and workplace culture, Different pre-understanding, Shifting premises and limited resources)	- Well, I don't think there is any difference between us, even if you are an AN or a RN or an anesthesiologist, there is no doctors' table in the staff room or anything like that (AN).<br>- It's very much a team effort/.../Actually, all staff categories are equally important. – But, of course, there is some kind of chain of command, so to speak (two anesthesiologists).<br>- There is a sectioning. The whole healthcare system consists of a hierarchy, unfortunately. /.../ it affects our work as well (RN).<br>- I manage the ICU mostly by myself. There is very little I discuss with the second-line manager (manager).<br>- That's something I miss a bit in our job, that we don't have so many forums where we meet (RN).<br>- I think we are quite used to change. I think it's because we work in the department we do. I think it's worse elsewhere (AN).<br>- Then, there are also different circumstances [affecting the change willingness] /.../ There is a lot of change here (RN).
**Processual circumstances** (Vague leadership, Few interprofessional meetings, Diffuse vision, Unequal information and staff involvement, Delayed follow-up, Enthusiasm, support and facilitation)	- To be able to say: Well, I have to leave at one o'clock, because I have a meeting with *XX and XX* [the anesthesiologist and the physiotherapist in the core project group] and some researcher, gives much more weight to my job /…/ suddenly, this is something really serious that we do [other agreeing] (member of core project group).<br>- Well, she [the first line manager] has supported us with time. Maybe it's difficult in other ways; she has her things to keep up with. The second-line manager isn't very interested. He wants the CP, I think… – Of course, they have other things to handle. There are many other issues as well (two core group members).<br>- I haven't interfered/.../ Maybe scheduled check-offs would have been beneficial/.../ We really didn't know the need, that's what we understand when looking back (manager).<br>- We thought we had a shared vision/.../and then someone read the draft and said: this isn't how we're going to do it, we don't have the time. Then the spirit was lost. And four months passed and nothing happened. Nobody knew if we were going to continue or not. Then, starting up after that was really hard (member of core project group).<br>- No, I have not actually done that [informed the anesthesiologists], I have to admit. Unfortunately, we have few forums to come together. We are currently a thin little crowd with big fallout over the last months (member of core project group).<br>- I do not think they [the anesthesiologists] are particularly informed. The information has mainly been directed at ANs and RNs, actually. We probably made a mistake. I haven't really thought of that (member of core project group).<br>- The guidelines were distributed before they were final, so you could have comments. – Almost every staff meeting we were informed, and there were education and reflection (two RNs).<br>- But now, I don't know if we have let it go somehow. We expect it to be implemented, and yet it's not (member of core project group).<br>- No, because I have other assignments /.../ I haven't followed it up as much as I wanted (member of core project group).

**Negotiating to achieve progress** (Role setting and working methods, Pathway modeling, Activity timing)	- Otherwise, I would have contributed more, but then I would have needed more time in the ICU (member of core project group).<br>- It's difficult to have time allocated an hour here and there. It's not enough to get into work (member of core project group).<br>- We had a vision in the beginning about an interprofessional approach, but we later understood that it wouldn't be possible. – Yes, there's been a lot of back and forth (two core group members).<br>- To learn, it's like you have to walk the mines yourself (member of core project group).<br>- The whole project took a long time, longer than planned. Sometimes it's impossible to get allotted time (RN).<br>- We got the guidelines successively, and then when the CP was completed, I felt that now everything we have is assembled (RN).<br>- There was a release party and then came summer. Later, when we were supposed to use it, we had forgotten what they said, sort of (AN).
**Output**	
**Varying utilization** (A nursing tool, Fluctuating documentation)	- Well, we maybe don't check the CP together. It's more like it is the RNs' administrative task in some way (AN).<br>- Still, we [the RNs] are using it and showing them. I cannot think of any anesthesiologist who by himself or herself is looking for a CP to follow (member of core project group).<br>- The interprofessional approach did not have any real impact (member of core project group).<br>- It's obvious that it [the CP] is the foundation, but it is not the most important thing for a new anesthesiologist to be aware of. It's more important to find the emergency room. – Well, it's important to know that you do not have to worry. There is a CP for patients on mechanical ventilation, so basically the nursing staff and physiotherapist manage the care on their own. So we actually have an information deficit (two anesthesiologists).<br>- I just think the anesthesiologists are not so involved in nursing, and it's mostly what the CP includes, how we take care of a patient on mechanical ventilation. They are rarely enthusiastic about that, they trust us to handle it. – Yes, they trust it will be done well and we are used to managing the care independently. We do not ask them about such things (two RNs).
**Improvements in understanding and practice** (Knowledge expansion, Clarified roles, Improved documentation, Care equality)	- In fact, it [the CP implementation] has meant a lot of things, partly because we have worked in a more structured way to further develop the care/.../ We are more certain that we have increased the basic quality level/.../ Before there were a lot more fluctuations, a lot more roller coaster in the care. Now it's a bit more equal. And you don't have to write as much, because everybody knows we follow the CP (manager).<br>- It has been educating/.../ to seek scientific literature, the library has been great. /.../ We have received a lot of knowledge, which we have implemented in the ICU (member of core project group).<br>- As when we worked with the guideline for sleep and discovered how much it included, just that little bit. You shouldn't just write about it, you should have all the references, and sit in the library and search for articles. – It was exciting (two ANs).<br>- There are more people saying and showing that they find the guidelines and read them (member of core project group).<br>- It's a foundation, some kind of key you can check [the CP]. Previously, you had to run and ask someone or look for answers/…/ This is much easier. You have it next to you to fall back on/.../ it is a quality assurance (RN).<br>- When you work with a new AN, I think the collaboration works much better [using the CP] /.../ everyone knows what to do, what is expected of you (RN).<br>- The same strategy applies so you control the lowest level of care, and the lowest level is quite high actually (anesthesiologist).<br>- I feel very proud of this and think: this is what we created, and it's useful if you want to use it (member of core project group).

Abbreviations: AN, assistant nurse; CP, clinical pathway; ICU, intensive care unit; RN, registered nurse

Note: * The quotes are from focus group and individual interviews and logbooks, different project phases and different individuals, but for confidentiality reasons specified only by staff category, manager, or member of the project's core group
