# Supplementary material for: Struggling for a feasible tool – the process of implementing a clinical pathway in intensive care: a grounded theory study
Source: BMC Health Serv Res. 2018 Nov 6;18:831. doi: 10.1186/s12913-018-3629-1 (PMC6219016; doi:10.1186/s12913-018-3629-1)
Supplement: Supplementary file 4 — Findings from the questionnaire. This file includes descriptive statistics from the questionnaire answered by staff in project phase four, about 1 year after implementation of the clinical pathway. (PDF 989 kb) [file 12913_2018_3629_MOESM4_ESM.pdf]

Additional File 4: Findings from the questionnaire. Descriptive statistics from the questionnaire answered by staff in project phase four, about one year after implementation of the clinical pathway. **Part 1**: Perceptions of working according to the CP; items sorted by areas presented by number (n) of informant and percentage (%) of each statement, as well as median score and intra quartile range (q^1–q^3) for the different staff categories in the four areas. **Part 2**: Additional questions, presented by number (n) of informants and percentage (%) of each statement, as well as the answers specified by staff category.

Part 1

**Items sorted by areas** (Highest to lowest possible score in the area)	**Totally agree** score 4	**Partly agree** score 3	**Partly disagree** score 2	**Totally disagree** score 1	**Registered nurses**	**Assistant nurses**	**Anesthesi-ologists**	**Total Sample**
					**Median score (q^1 q^3)** / number (%) totally or partly agree			
**Usability** (score 5–20) n=38 [1]	n (%)	n (%)	n (%)	n (%)	**17 (15–18)** n = 17	**15 (14–18)** n = 16	**15 (14–16)** n = 5	**16 (14–18)** n = 38
I feel that it is easy to work according to the CP	13 (34)	23 (61)	2 (5)	–	17 (100)	16 (100)	4 (80)	36 (95)
It is easier and quicker to read and get knowledge about a patient who is cared for according to the CP than a patient without the CP	12 (32)	17 (45)	5 (13)	3 (8)	13 (76)	12 (75)	5 (100)	29 (76)
It is easier to see which nursing measures have been taken when the patient has the CP than without the CP	13 (34)	16 (42)	8 (21)	1 (3)	11 (65)	14 (88)	5 (100)	29 (76)
The use of the CP helps me structure my work so that nothing gets forgotten	14 (37)	18 (47)	5 (13)	1 (3)	15 (88)	14 (88)	4 (80)	32 (84)
I feel that it is easy to work with the CP at our ward	14 (37)	21 (55)	3 (8)	–	16 (94)	15 (94)	5 (100)	35 (92)
**Documentation** (score 5–20) n=38 [1]	n (%)	n (%)	n (%)	n (%)	**16 (16–18)** n = 17	**15 (14–17)** n = 16	**15 (13–17)** n = 5	**16 (14–18)** n = 38
I feel that it is quicker to document when the CP is used than without the CP	18 (47)	16 (42)	2 (5)	1 (3)	16 (94)	13 (81)	4 (80)	34 (89)
I feel it is easy to document when the CP is used	15 (39)	18 (47)	2 (5)	–	16 (94)	13 (81)	4 (80)	33 (87)
I feel that double documentation has decreased with the use of the CP	13 (34)	21 (55)	2 (5)	1 (3)	16 (94)	14 (88)	4 (80)	34 (89)
It is easier to see what needs to be done when the patient has the CP than if documentation consists of running notes	18 (47)	15 (39)	2 (5)	2 (5)	15 (88)	14 (88)	4 (80)	33 (87)
My methods of documentation become restricted when I work according to the CP	4 (11)	10 (26)	12 (32)	10 (26)	*9 (53)*	*3 (19)*	*2 (40)*	*14 (37)*
**Quality of care** (score 9–36) n=38 [1]					**28 (26–31)** n = 17	**27 (25–32)** n = 16	**28 (22–28)** n = 5	**27 (25–31)** n = 38
I feel that I have more time for patient-focused care as the CP was implemented	1 (3)	21 (55)	12 (32)	3 (8)	9 (53)	1 (6)	3 (60)	24 (63)
I get a quick overall view about the patient's condition when using the CP	10 (26)	19 (50)	5 (13)	3 (8)	13 (76)	13 (81)	3 (60)	29 (76)
I feel that patient cared for according to the CP receive good and safe care to a greater extent	14 (37)	16 (42)	5 (13)	2 (5)	13 (76)	13 (81)	4 (80)	30 (79)
The knowledge overview related to the CP has been helpful for me to understand why we should care for the patient in accordance with the measures in the CP	16 (42)	17 (45)	3 (8)	1 (3)	15 (88)	15 (94)	3 (60)	33 (87)
I do not deviate from the CP unless the patient's condition necessitates an individual care plan	9 (24)	15 (39)	8 (21)	3 (8)	13 (76)	11 (69)	–	24 (63)
I have greater trust in my own work experience than in the content of the CP	1 (3)	12 (32)	18 (47)	6 (16)	*4 (24)*	*6 (38)*	*3 (60)*	*12 (32)*
It is easier for new staff to care for a patient with the CP than without the CP	24 (63)	11 (29)	3 (8)	–	16 (94)	14 (88)	5 (100)	35 (92)
The use of the CP facilitates the introduction of new staff	22 (58)	14 (37)	2 (5)	–	17 (100)	14 (88)	5 (100)	36 (95)
I was informed that the knowledge overview related to the CP is supposed to be based on evidence	20 (53)	12 (32)	3 (8)	1 (3)	15 (88)	13 (81)	4 (80)	32 (84)
**Implementation process** (score 4–16) [2]					**11 (9–13)** n = 9	**12 (10–13)** n = 9	**15** [3] n = 1	**12 (9.5–13)** n = 19
I was well aware of what a CP is when it was time to start using it	6 (32)	11 (58)	1 (5)	1 (5)	7 (78)	9 (100)	1	17 (89)
I continuously received information about the work on developing the CP	5 (26)	10 (53)	3 (16)	1 (5)	6 (67)	8 (89)	1	15 (79)
I felt well informed about the expected benefits of using the CP	5 (26)	8 (42)	5 (26)	1 (5)	5 (56)	7 (78)	1	13 (68)
I felt that I participated in the work on developing the CP	4 (21)	6 (32)	6 (32)	3 (16)	4 (44)	5 (56)	1	10 (53)

Notes: CP: Clinical pathway. [1)] The differences between total n and the sum of statements represent non-responses. [2)] Includes only participant working in the setting since start of the project. [3)] Only one participant/response. [4)] The differences between total n and the sum of statements represent the response 'not utilized'. Writing in italics represents negatively asked questions thereby reversed in analysis.

Continued on next page

Part 2

**Additional questions**	**Totally agree** n (%)	**Partly agree** n (%)	**Partly disagree** n (%)	**Totally disagree** n (%)	**Registered nurses**	**Assistant nurses**	**Anesthesi-ologists**	**Total Sample**
Strategies of importance to achieving successful implementation of the CP (several alternatives could be selected) n=24 [2,4]					**Number (%) totally or partly agree**			
					n = 12	n = 10	n = 2	n = 24
Internal facilitator	14 (58)	7 (29)	–	1 (4)	12 (100)	8 (80)	1 (50)	21 (87)
Training prior to implementation	13 (54)	8 (33)	1 (4)	–	12 (100)	8 (80)	1 (50)	21 (87)
Reminders after implementation	14 (58)	6 (25)	2 (8)	–	12 (100)	7 (70)	1 (50)	20 (37)
Ongoing training	7 (29)	11 (46)	1 (4)	–	11 (92)	6 (60)	1 (50)	18 (75)
Written information	8 (33)	7 (29)	3 (12)	1 (4)	9 (75)	5 (50)	1 (50)	15 (62)
Computerized support system	5 (21)	8 (33)	3 (12)	–	8 (67)	4 (40)	1 (50)	13 (54)
External facilitator	1 (4)	12 (50)	1 (4)	2 (8)	7 (58)	5 (50)	1 (50)	13 (54)
Regular feedback	5 (21)	8 (33)	5 (21)	–	8 (67)	5 (50)	–	13 (54)
Feedback and evaluation on one occasion	4 (17)	8 (33)	4 (17)	–	7 (58)	5 (50)	–	12 (50)
	**Yes**		**No**		**Number (%) stating successful implementation**			
Do you perceive the implementation of the CP to be successful? [2]					n = 12	n = 10	n = 1	n = 23
	19 (79)		4 (17)		9 (75)	9 (90)	1 [3]	19 (79)
	**Always**	**Often**	**Seldom**	**Never**	**Number (%) stating CP is Always/Often utilized**			
When you care for a patient with mechanical ventilation, how often do you perceive the CP is utilized?					n = 20	n = 17	n = 6	n = 43
	9 (21)	32 (74)	2 (5)	–	19 (95)	16 (94)	6 (100)	41 (95)
	**Participate in decision**	**Informed CP is used**	**Neither**	**I do not know**	**Number (%) stating patient/family Participate or are Informed**			
					n = 19	n = 18	n = 6	n = 43
In what way are the patients involved in the decision to use the CP (when their condition allows)?	2 (5)	7 (16)	33 (77)	1 (2)	2 (11)	3 (17)	4 (67)	9 (21)
In what way is family/next of kin involved in the decision to use the CP?	2 (5)	8 (19)	28 (65)	5 (11)	3 (16)	3 (17)	4 (67)	10 (23)
Which categories of staff utilize the CP?					**Number (%) stating the category uses the CP**			
					n = 20	n = 16	n = 6	n = 42
Registered nurses					19 (95)	15 (94)	6 (100)	40 (95)
Assistant nurses					18 (90)	13 (81)	4 (67)	35 (83)
Anesthesiologists					6 (30)	11 (69)	5 (83)	22 (53)
Physiotherapist					5 (25)	8 (50)	–	13 (31)
Physicians from other clinics					–	–	–	–
What is you general impression of the CP?					**Number (%) positive to the CP**			
**Positive** It facilitates my work/ **Negative** It hinders my work					16 (100)	16 (100)	5 (100)	37 (100)

Notes: CP: Clinical pathway. [1)] The differences between total n and the sum of statements represent non-responses. [2)] Includes only participant working in the setting since start of the project. [3)] Only one participant/response. [4)] The differences between total n and the sum of statements represent the response 'not utilized'. Writing in italics represents negatively asked questions thereby reversed in analysis.
